# Patients' memories from intensive care unit: A qualitative systematic review

**DOI:** 10.1002/nop2.804

**Published:** 2021-02-21

**Authors:** Charlotte C. Maartmann‐Moe, Marianne Trygg Solberg, Marie Hamilton Larsen, Simen A. Steindal

**Affiliations:** ^1^ Lovisenberg Diaconal University College Oslo Norway; ^2^ Emergency Department Oslo University Hospital Oslo Norway

**Keywords:** critical care, critical illness, memories, nursing, qualitative, recollections, systematic review

## Abstract

**Aim:**

To identify and synthesize the evidence regarding adult patients' memories from their stay in the intensive care unit.

**Design:**

A qualitative systematic review and meta‐synthesis. PROSPERO # CRD42020164928. The review employed the guideline of Bettany‐Saltikov and McSherry and the Enhancing transparency in reporting the synthesis of qualitative research guidelines.

**Methods:**

Systematic search for qualitative studies published between January 2000 and December 2019 in Cumulative Index to Nursing and Allied Health, Medical Literature Analysis and Retrieval System Online, PsycINFO, and Excerpta Medica Database. Pairs of authors independently assessed eligibility, appraised methodological quality using Joanna Briggs's quality appraisal tool and extracted data. The analysis followed the principles of interpretative synthesis.

**Results:**

Sixteen papers from 15 studies were included in the review. Three themes emerged: (a) memories of surreal dreams and delusions, (b) care memories from sanctuary to alienation and (c) memories of being vulnerable and close to death.

## 1 INTRODUCTION

Patients admitted to the intensive care unit (ICU) are critically ill and often require life‐sustaining treatment, technological support and continuous monitoring as well as emotional support. A stay in the ICU can be distressing and wearing on the patients (Adhikari et al., 2011; Rose et al., 2011). The lifesaving and highly complex ICU environment may cause patients to encounter multiple stressors, such as being confronted with own mortality, unfamiliar noises, invasive procedures, pain, sedation, delirium and inability to speak (Burki, 2019). Hence, the patients' illness and the medical care provided may alter their ability to comprehend and take in their surroundings.

Memories are defined as the minds ability to take in information, store it and recall it at a later time (Zlotnik & Vansintjan, 2019). Short‐term memory refers to information processed in a limited period of time, while long‐term memory allows us to store information for extended periods, including information that can be retrieved consciously or unconsciously (Camina & Güell, 2017). Hence, a memory is the imprint an experience has created in our mind, although it should be understood that the memory has been moulded in our mind and can be influenced and changed (Redelmeier & Kahneman, 1996).

Previous studies have shown that patients have a range of distressing memories from their ICU stay. Three types of ICU memories have been reported: factual, emotional and delusional memories (Samuelson et al. 2008). Factual memories are common, such as memories of ventilator treatment and other procedures, which are reported by 20%–83% of prior ICU patients (Magarey & McCutcheon, 2005; Ringdal et al., 2006; Roberts et al., 2007). Correspondingly, delusional memories such as hallucinations and dreams are reported by 21%–73% of ICU patients (Ringdal et al., 2006; Rundshagen et al., 2002). Furthermore, patients may recall pain and anxiety, as well as a feeling of being safe (Egerod et al., 2015; Stein‐Parbury & McKinley, 2000. Studies show that delusional memories are an important causative factor for developing symptoms of posttraumatic stress disease (PTSD) postdischarge, such as anxiety and depression (Morrissey & Collier, 2016; Jones et al., 2001; Wu et al., 2018).

### 1.1 Background

A meta‐synthesis explored patients' difficulties during critical illness, describing how patients' suffering was altered in relation to sedation protocols (Egerod et al., 2015). Another qualitative review disclosed how patients changed their perception of what was real and unreal during their critical illness, as well as experiencing proximity to death (Cutler et al., 2013). Other reviews have investigated how the ICU affects patients, exploring the prevalence of PTSD in ICU survivors (Davydow et al., 2008), and in relation to delusional memories (Kiekkas et al., 2010). Recall of delusional memories after ICU discharge seems to be associated with PTSD‐related symptoms (Kiekkas et al., 2010). An integrated review including quantitative studies examined the relationship between ICU treatment and memories after discharge in ICU survivors. The results suggest that deep sedation and administration of corticoids contribute to delusional memories (Aitken et al., 2016).

Furthermore, a literature review investigated patients' memories from the ICU exploring patients' general impressions of their stay found that patients recalled positive, neutral and negative experiences (Stein‐Parbury & McKinley, 2000).

To the best of our knowledge, no recent qualitative systematic review (SR) has been conducted to synthesize patients' memories of their ICU stay. Such a review could enhance healthcare professionals' understanding of patients' memories of their ICU experiences. By gaining insight into what characterizes patients' memories, healthcare professionals could be further equipped to tailor care on the basis of updated knowledge of patients' recalled experiences from the ICU.

## 2 METHODS

### 2.1 Aim

The aim of this systematic review was to identify and synthesize the evidence regarding adult patients' memories from their stay in the ICU.

### 2.2 Design

The qualitative SR was guided by the guideline for SRs described by Bettany‐Saltikov and McSherry (2016), which consists of seven steps: (a) formulating an answerable and focused review question; (b) specifying objectives, inclusion and exclusion criteria; (c) conducting a comprehensive and systematic literature search; (d) selecting the studies to include in the review; (e) appraising the methodological quality of the included research papers; (f) extracting the data; and (g) synthesizing, summarizing and presenting the findings. The review was also guided by the “Enhancing transparency in reporting the synthesis of qualitative research (ENTREQ)” guidelines (Tong, Flemming, McInnes, Oliver, & Craig, 2012). The analysis followed the principles of interpretative synthesis. The review was registered in PROSPERO (registration number: CRD42020164928).

### 2.3 Inclusion and exclusion criteria

Studies published in English, Swedish, Danish or Norwegian language in peer‐reviewed journals were included if they met the following criteria: (a) they had a qualitative design, (b) collected data using interview, (c) they included patients submitted to the ICU for 24 hr or more, regardless of diagnosis, and (d) reported patients' memories, recollections or similar concepts from the ICU. Studies were excluded if (a) patients were submitted for less than 24 hr or if not reported, (b) patients were aged 17 years or younger, (c) if proxy reporting by healthcare professionals or next of kin were used and (d) the data were published as letters, comments, conference abstract, doctoral thesis or as any type of review.

### 2.4 Search methods

A systematic and comprehensive literature search for qualitative studies published between 1 January 2000 and 16 December 2019 was conducted in the following databases on 16 December 2019: Cumulative Index to Nursing and Allied Health (CINAHL), Medical Literature Analysis and Retrieval System Online (MEDLINE), PsycINFO and Excerpta Medica Database (EMBASE). The timeframe of publications included was considered by the authors to be a period where the ICU medicine was changing and advancing, and studies published before 2000 were considered to be less relatable to the ICU‐care provided after the millennium. The search strategy was built in CINAHL by the first author and an experienced librarian. The review question was deconstructed, identifying the component parts in a PEO‐table, sorting population (P), exposure (E) and outcome (O). The components were paraphrased into searchable terms and text words and are shown in Table 1. The search conducted in CINAHL is shown in Table 1 and was later adapted to the other databases. A manual search, screening the reference list of reviews and relevant studies, was also conducted.

**TABLE 1:** Search strategy from Cumulative Index to Nursing and Allied Health (CINAHL), organized according to the Population, Exposure, Outcome (PEO) table

PEO table	Search number	CINAHL headings, text word and combinations
Population (P)	1	(MH “Critically Ill Patients”)
	2	critical* n3 care n3 patient*
	3	critical* n3 ill n3 patient*
	4	critical* n2 ill
Exposure (E)	5	(MH “Intensive Care Units”)
	6	ICU
	7	critical* care n3 unit
	8	intensive n3 care n3 unit
Outcome (O)	9	(MH “Memory+”)
	10	memor*
	11	recollection*
	12	recall*
	13	remember*
	14	experience*
	15	1 OR 2 OR 3 OR 4
	16	5 OR 6 OR 7 OR 8 OR
	17	9 OR 10 OR 11 OR 12 OR 13 OR 14
	18	15 AND 16 AND 17

### 2.5 Study selection

**FIGURE 1:**
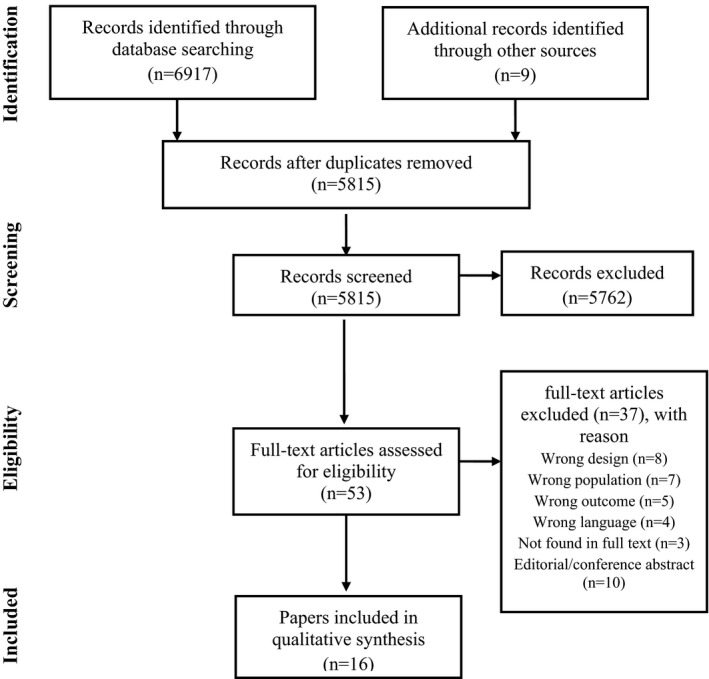
Flow diagram showing numbers of studies included and excluded

On the basis of the inclusion and exclusion criteria, two pairs of authors independently screened papers for inclusion. Rayyan, a web tool that helps expedite the initial screening of abstracts and titles, was used to facilitate the publication selection as well as blinding (Ouzzani et al., 2016). Publications were first considered for inclusion on the basis of title and abstract. The possible eligible studies were then examined by assessing the full text. When there was any doubt whether a publication should be included or not, a third author independently assessed the publication. The database search and the manual search gave a total of 6,926 papers, and the titles and abstracts of 5,815 publications were screened. The full text of 53 publications was read, and the final sample consisted of 15 studies, presented in 16 papers (see Figure 1). The reasons for exclusion of papers are show in Figure 1.

### 2.6 Data extraction

The first author extracted data from the included papers using a standardized data collection form, while the information was checked by the last author. Conflicts were resolved by consensus or in consultation with a third author. The data collection form included the following information: author, country and year of publication, aim, sample size and characteristics, design and method, time of data collection, and main findings related to our aim. In addition, key quotes from the findings section of the included papers were extracted for the qualitative content analysis.

### 2.7 Quality appraisal

The quality of the included papers was independently assessed by pairs of authors, using the Joanna Briggs Institute appraisal tool for qualitative research (Lockwood et al., 2015). The results of the quality appraisal are shown in Table 2. No papers were excluded on the basis of the results of the quality appraisal.

**TABLE 2:** Critical Appraisal Checklist for Qualitative Research for the included articles

Authors	Congruity between philosophical perspective & methodology?	Congruity between methodology & research question/ objectives?	Congruity between the research methodology & methods used to collect data?	Congruity between methodology & representation & data analysis?	Congruity between research methodology & result interpretation?	Locating the researcher culturally or theoretically?	Influence of the researcher on the research, & vice‐ versa, addressed?	Participants, and their voices, adequately represented?	Ethical considerations?	Conclusions drawn flow from the analysis, or interpretation, of the data?	Total number of yes
Adamson et al. (2004)	N	Y	Y	Y	Y	N	N	Y	Y	Y	7/10
Alexandersen et al. (2019)	Y	Y	Y	Y	Y	Y	Y	Y	Y	Y	10/10
Ballard et al. (2006)	Y	Y	Y	Y	Y	Y	Y	Y	Y	Y	10/10
Karlsson and Forsberg (2008)	Y	Y	Y	Y	Y	Y	Y	Y	Y	Y	10/10
Laerkner et al. (2017)	Y	Y	Y	Y	Y	U	Y	Y	Y	Y	9/10
Löf et al. (2006)	N	Y	Y	Y	Y	Y	Y	Y	Y	Y	9/10
Löf et al. (2008)	N	Y	Y	Y	Y	Y	Y	Y	Y	Y	9/10
Magarey and McCutcheon (2005)	N	Y	Y	Y	Y	Y	N	Y	Y	Y	8/10
Meriläinen et al. (2013)	N	Y	Y	Y	Y	N	N	Y	Y	Y	7/10
Minton and Carryer (2005)	N	Y	Y	Y	Y	Y	N	Y	Y	Y	8/10
Olsen et al. (2017)	N	Y	Y	Y	Y	N	N	Y	Y	Y	7/10
Page et al. (2019)	Y	Y	Y	Y	Y	Y	Y	Y	Y	Y	10/10
Pattison et al. (2007)	Y	Y	Y	Y	Y	N	N	Y	Y	Y	8 / 10
Roberts and Chaboyer (2004)	N	Y	Y	N	N	N	N	N	Y	Y	4/10
Storli et al. (2008)	Y	Y	Y	Y	Y	N	Y	Y	Y	Y	9/10
Tembo et al. (2012)	Y	Y	Y	U	U	U	Y	Y	Y	U	6/10

Note

Y: Yes, N: No, U: Unclear/

### 2.8 Data abstraction and synthesis

For this systematic review, the data abstraction was conducted in line with the principles of an interpretative synthesis (Dixon‐Woods et al., 2005). The analyses were guided by inductive qualitative content analysis as described by Graneheim and Lundman (2004), which has been used in previous qualitative SRs (Eriksen et al., 2020; Sibbern et al., 2017; Uhrenfeldt et al., 2013). Conducting a qualitative content analysis gives the opportunity to interpret manifest and as well as latent content (Graneheim et al., 2017).

The findings section of each included paper was read several times to obtain a sense of the material as a whole. The text describing patients' memories from the ICU stay was identified and studied further. Meaning units that were considered to relate to and illuminate the aim were highlighted and extracted from the results section of the included papers. These meaning units were condensed by shortening the text while preserving its core. The condensed meaning units were abstracted by coding and categorizing the material. Codes were created to describe and interpret the condensed meaning units. The codes generated across the studies were compared according to differences and similarities and gathered into categories to unite the data and conjugate the findings. Categories considered to be related to each other were grouped together. Guided by our aim and through discussions among all the authors, the underlying meanings in these categories were abstracted and interpretated into new themes across the papers. This enabled the analysis to go beyond the content of the included papers. An example of data abstraction and synthesis process is shown in Table 3.

**TABLE 3:** Example of the analysis process

Meaning unit	Condensed meaning unit	Code	Category	Theme
“Patient E spoke at 3 months of fear and panic related to not being able to breath in connection with weaning from the ventilator”	Remember feeling panic about not being able to breath when weaning from the respirator	Panic when not being able to breath on the ventilator	Feeling scared and life threatened	Memories of being vulnerable and close to death
‘… I was in the Recovery Room and then there was this terrible pain shot up my back and hit my head and it just kept exploding and I just screamed… The pain just exploded. I'll never forget it… The pain was so horrific.’	Terrible pain shooting through me, it was exploding, and I kept screaming. It was horrific	Remembering horrific pain		
“Despite the fact that he had no grasp of what was going on or why he was in intensive care, he felt with his entire body that his life was threatened, that it was all over”	No grasp of what was happening, he felt in his body how his life was threatened	Feeling in body how life was threatened		
“Others described the distress of not being able to sleep or move properly”	Distressed about not being able to move or sleep properly	distressed about not being able to move or sleep	Feeling helpless and in loss of control	
“I remember them giving me medicine. That's like all I remember. More drugs. Them messing with me. I could hear them talking and then putting more stuff in me.”	Them giving me medicine, feeling drugged, them messing with me, putting more stuff in me	Loss of control when feeling drugged		
“And it's quite strange because I couldn't speak to them or I couldn't get anything… yeah, like I couldn't say “Hey it's me over here”. I couldn't say that. And I felt like I was paralysed to the bed”.	Not able to speak, not able to ask for attention, feeling paralysed	Feeling helpless when not able to communicate		

## 3 FINDINGS

### 3.1 Characteristics of the included studies

The included studies were conducted in Sweden (*N* = 2), Australia (*N* = 4), Norway (*N* = 3), the United States (*N* = 1), Denmark (*N* = 1), Finland (*N* = 1), New Zealand (*N* = 1) and the United Kingdom (*N* = 2). The included studies were published between 2004–2019. The sample size of the included studies ranged from 4–31 participants and included a total of 216 participants. The participants' age ranged from 19–86 years, while length of stay ranged from 1 day to 40 days. The studies used grounded theory (*N* = 3), hermeneutic phenomenological (=3), phenomenological (*N* = 2), descriptive (*N* = 4), explorative (*N* = 1), interpretive description (*N* = 1) or mixed method (all data were collected using unstructured in‐depth interviews; *N* = 1) approach. In all the studies, data were collected using individual interviews. The time of the data collection ranged from 48 hr after extubation to 10 years after the ICU stay. The characteristics of the included studies are shown in Table 4.

**TABLE 4:** Characteristics of the included studies

Author, year country	Aim	Sample size and characteristics	Design and method	Time of data collection	Findings
Adamson et al., (2004) Australia	To examine the participant’ memories of intensive care and hospitalization at 6 mounts post‐discharge, and to explore the impact of the critical illness experience on the recovery	6 participants, 4 males, aged 57–83 years. Submitted to the ICU for at least 48 hr	Strauss and Corbin's grounded theory approach. Semi‐structured interview Qualitative content and thematic analysis	6 months after ICU	Memories of comforts and discomforts
Alexandersen et al. (2019) Norway	To retrospectively explore the experiences of inner strength and willpower among long‐term ICU patients throughout their illness trajectory. The study aimed at a deeper understanding of aspects that promote or challenge long‐term ICU patients' inner strength and willpower	17 participants, 4 females, 13 men, average age 55.2 (27–76 years) years, submitted to the ICU for 7 days or longer	Qualitative hermeneutic‐phenomenological approach In‐depth interviews	6–20 months after ICU discharge	Positive dreams and terrifying delusions influenced the patients
Ballard et al. (2006) USA	To determine and describe the remembered experiences of critical care patients who were given neuromuscular blocking agents and sedatives and/or analgesics to facilitate mechanical ventilation, improve haemodynamic stability, and improve oxygenation	11 participants, 4 males, aged 19–69 years. NMBA via continuous infusion minimum of 6 hr. Spent at least 48 hr on a ventilator	Phenomenological approach Bedside unstructured interview Constant comparative analysis	48–72 hr after extubation	Themes that emerged; Back and forth between reality and the unreal, Between life and death
Karlsson and Forsberg (2008) Sweden	To investigate experiences of being conscious during ventilator treatment in the ICU from a patient's perspective	8 participants, 4 males, aged 21–81 years. Ventilator treatment from one day to several months	Hermeneutic, phenomenological approach In‐depth, unstructured interviews Interpretative and thematic analysis	NR	Themes that emerged: Memories, Mastering one's situation, A renewed me, and Confirmation
Laerkner et al. (2017) Denmark	To explore patients' experiences of being awake during critical illness and MV in the ICU	22 participants, 14 males, aged 50–86 years, submitted to the ICU for at least 72 hr	Interpretive description, inductive, qualitative approach inspired by ethnography, grounded theory and phenomenology Thematic analysis	First interview during the first week after ICU discharge, a second interview after 2–4 months	Memories of the surrounding activities and feeling powerless when ignored by the staff and being affected when witnessing fellow patients' suffering
Löf et al. (2006) Sweden	To describe critically ill and ventilator‐treated patients' recollections of both factual events and unreal experiences at 3 and 12 months postdischarge from ICU	9 participants, 6 males, aged 42–77 years, ventilator treated for more than 3 days, average ICU stay 24 days	Qualitative design Qualitative content analysis and continuous comparative analysis	3 and 12 months postdischarge from ICU	Recall of unreal experiences, Recall of factual events, Fragmentary memory of factual events, No recall of factual events
Löf et al. (2008) Sweden	To describe ICU patients' recall of their emotional reactions, from falling critically ill to hospital discharge; this at 3 and 12 months after discharge from the ICU	9 participants, 6 males, aged 42–77 years. Ventilator treated for more than 3 days, average ICU stay 24 days	A descriptive design Face‐to‐face interviews, based on a written guide with open questions minutes Qualitative content analysis	3 and 12 months postdischarge from the ICU	Results presented as sub‐themes: Feelings of comfort/discomfort, Bodily sensations, existential threat and managing these experiences
Magarey and McCutcheon (2005) Australia	To explore the memories of patients who had a short‐term admission to the ICU, with a particular focus on dreams, nightmares and confusion	8 participants, 6 males, aged 34–84 years. Submitted to the ICU 24 hr or more	Descriptive design Face‐to‐face, semi‐structured open‐ended interviews Thematic analysis	NR	Emerging themes: Reality and unreality, Blackness and colour, Powerlessness and purpose, Death
Meriläinen et al. (2013) Finland	To describe the interaction between intensive care patients and the ICU environment from the perspective of the hospital bed and patients' memory of the ICU	4 patients, 3 males, aged 20 to 45 years. Mean stay in ICU 13.5 days	Mixed methods design Unstructured in‐depth interviews Inductive and deductive content analysis	Interviewed 3and 6 months postdischarge from ICU	Two categories emerged: Memories of internal experiences, Memories of external experiences
Minton and Carryer (2005) New Zealand	To describe the memories of former ICU patients	6 participants. Sex NR, age NR, submitted to the ICU for over 24 hr	Descriptive design Semi‐structured interviews Thematic analysis	6 months postdischarge from ICU	Themes that emerged: Loss of control and dependence on technology, Distorted thoughts, memories of procedures, proximity to death, moving on
Olsen et al. (2017) Norway	To investigate how adult patients experience their intensive care stay, their recovery period, and usefulness of an information pamphlet	29 participants, 19 males, aged 20–80 years, MV for 48 hr	Exploratory design Semi‐structured interviews Qualitative content analysis	3 months postdischarge from hospital	Two themes emerged: Being on an unreal, strange journey, Normalizing the abnormal
Page et al. (2019) The UK	To understand the critical illness trajectory from patients and relative perspective	16 participants (patients), 10 males, median age 61 (42–75 years) years, length of stay 4–40 days	Constructivist grounded theory methodology In‐depth‐interviews Constant comparative analysis and data collection	Interviews 4–10 days after discharge	Experienced unusual, recurring dreams and/or hallucinations or nightmares
Pattison et al. (2007) The UK	To establish patients' experiences after discharge form critical care and to evaluate implementation of a follow‐up service	27 participants, aged 18 years or older, spent at least 48 hr in the ICU	Prospective, longitudinal, exploratory study In‐depth, unstructured interviews Grounded theory approach	Interviewed at 3 and 6 months postdischarge from ICU	Memories of real and unreal experiences, disorientation
Roberts and Chaboyer (2004) Australia	To describe the patients' subjective experiences of dreaming using patient interviews at 12–18 mounts after ICU discharge and to examine the relationship between these reports and the patients observed behaviour (delirium/no delirium) while in the ICU	31 participants, 23 males, aged around 60 years, admitted to ICU for more than 72 hr, average stay of about 1 week	Descriptive study design Semi‐structured Interviews A mid‐range accounting scheme was used in the analysis	12 to 18 months after discharge from the ICU	Findings presented as types of dreams
Storli et al. (2008) Norway	To explore the meaning of living with memories from intensive care	10 participants, 4 males, aged 28–70 years, spent minimum 4 days on artificial ventilation	A hermeneutic‐phenomenological approach In‐depth interview	10 years after ICU stay	Categories that emerged; Looking back, The journey, 10 years later
Tembo et al. (2012) Australia	To explore the experience of critically ill patients in ICU and beyond	12 participants, participant characteristics NR	Phenomenological approach In‐depth interviews Hermeneutic‐phenomenology thematic analysis using van Manen's six dynamic interplay activities	First interview 2 week after ICU discharge, second interview after 6–11 months	Memories of being “absent” from the world and a feeling of being imprisoned and trapped by the ICU and ICU therapies. Participants experienced restriction and threat to their life during the wakefulness phase of their ICU stay

Abbreviations: h, hours; ICU, intensive care unit; MV, mechanical ventilated; NMBA, neuromuscular blocking agent; NR, not reported; UK, United Kingdom; US, United States.

The data synthesis revealed three new themes: (a) memories of surreal delusions and dreams, (b) care memories from sanctuary to alienation and (c) memories of being vulnerable and close to death (Table 5).

**TABLE 5:** Overview of the themes and categories

Themes	Categories
Memories of surreal dreams and delusions	Memories of total confusion
	Recollecting dreams and nightmares
	Recollecting delusions and hallucinations
Care memories from sanctuary to alienation	Being provided good care, nurses providing safety and security
	Feeling treated like an object, feeling needs being disregarded by nurses
	Experiencing an imbalance of power
Memories of being vulnerable and close to death	Feeling helpless and loss of control
	Feeling scared and life being threatened

### 3.2 Memories of surreal dreams and delusions

In the majority of the studies, patients described having surreal recollections, which many described as the most distinct and scary. The ICU stay was remembered as a time of confusion and disorientation and many did not know where they were. Patients described being in a state of constant haziness, not knowing if they were awake or asleep and unable to separate day and night. Patients described the border between the real and unreal as being blurred (Adamson et al., 2004; Löf, Berggren, & Ahlström, 2006, 2008; Meriläinen et al., 2013; Olsen et al., 2017; Pattison et al., 2007; Storli et al., 2008). This led them into a chaotic state of mind: “They found it difficult to differentiate between memories of ICU, the ward and their dreams and nightmares” (Minton & Carryer, 2005).

Patients had several memories encompassing a wide range of dreams, including travelling, the nurses and peculiar people that were out of the ordinary, not necessarily causing fear. These dreams were often described in detail and were perceived as real (Guttormson, 2014; Karlsson & Forsberg, 2008) emphasizing the patients' degree of confusion and disorientation. One patient dreamt that he was physically connected to things (Karlsson & Forsberg, 2008), and others had dreams where family members portrayed hospital staff (Roberts & Chaboyer, 2004). Furthermore, patients recalled comforting or inspiring dreams with relatives or godly figures coming to their aid (Alexandersen et al., 2019; Magarey & McCutcheon, 2005).

The majority of patients' recollected dreams were nightmares. Some recalled unpleasant and scary dreams (Pattison et al., 2007; Roberts & Chaboyer, 2004). Several described nightmares as terrifying (Alexandersen et al., 2019; Löf et al., 2006). Patients had nightmares about being locked up or restrained, trying to escape (Karlsson & Forsberg, 2008; Löf et al., 2006), about dying (Magarey & McCutcheon, 2005), or that nurses were trying to harm or kill them (Page et al., 2019; Roberts & Chaboyer, 2004). One patient described how “The dreams always involved feelings of being caught in a situation, being ill and trapped in, for example, a black hole” (Karlsson & Forsberg, 2008).

A persistent recollection, represented across the studies, was of patients' upsetting hallucinations and delusions. Several patients saw things such as insects and animals in the room (Löf et al., 2006; Storli et al., 2008), and some saw things emerging through the walls (Adamson et al., 2004; Magarey & McCutcheon, 2005) or blood on the surfaces of the room (Löf et al., 2006). Patients reported having paranoid hallucinations of people trying to hurt and kill them, including nurses (Löf et al., 2006; Minton & Carryer, 2005; Olsen et al., 2017). Some had delusions of being surrounded by dead bodies (Storli et al., 2008). Common to these hallucinations was that they evoked fear, and several described them as horrifying. Furthermore, patients commonly described memories of hallucinations that reoccurred, seemed realistic and were intimidating (Löf et al., 2008; Roberts & Chaboyer, 2004). However, some had unthreatening visions which even brought forth delight and could consist of calming colours in the room (Magarey & McCutcheon, 2005; Olsen et al., 2017; Roberts & Chaboyer, 2004).

### 3.3 Care memories from sanctuary to alienation

In the majority of the papers, patients described memories of the care they were provided in the ICU. Patients recalled nurses surrounding them, performing procedures on them and their fellow patients (Löf et al., 2006; Meriläinen et al., 2013). For many patients, the nurses especially evoked emotions of safety and security. The nurses were remembered by their warm touch, kind voices, reassuring words and as being technically competent and by their ability to collaborate and treat them as a person (Adamson et al., 2004; Laerkner et al., 2017; Löf et al., 2006, 2008; Olsen et al., 2017). Patients remembered nurses relieving their discomforts by removing secretions from their airways and alleviating their pain (Adamson et al., 2004; Karlsson & Forsberg, 2008).

Patients' interactions with nurses were not remembered as exclusively reassuring, some had memories of nurses making them feel helpless, like they were not seen or heard (Karlsson & Forsberg, 2008; Laerkner et al., 2017; Meriläinen et al., 2013). Others described feelings of being left alone and that the nurses did not have time for them (Laerkner et al., 2017; Olsen et al., 2017). The patients described times where they felt the nurses were annoyed and perceived that the nurses got tired of them and their needs (Karlsson & Forsberg, 2008; Löf et al., 2008; Magarey & McCutcheon, 2005; Meriläinen et al., 2013). “I don't remember being in pain because the nurses would come… maybe to keep me quiet” (Adamson et al., 2004). Furthermore, patients remembered nurses discussing private issues and other patients while caring for them (Laerkner et al., 2017; Löf et al., 2006; Minton & Carryer, 2005). “I experienced a time when it [for the nurses] was more about talking to each other than taking care of me. I was so annoyed” (Laerkner et al., 2017).

Patients remembered distressing disturbances from their surroundings and described constant chaos of sound, light and a flow of people (Alexandersen et al., 2019; Karlsson & Forsberg, 2008; Löf et al., 2006; Olsen et al., 2017). Also, they recalled hearing suffering fellow patients and seeing procedures being performed on other patients (Karlsson & Forsberg, 2008; Laerkner et al., 2017; Meriläinen et al., 2013; Minton & Carryer, 2005). Patients described vivid memories of unpleasant procedures (Minton & Carryer, 2005; Olsen et al., 2017); several were described as horrible. “I remember them shoving that thing down my throat. She [the nurse] said, ‘Just swallow, just like handle it’ I was choking. It was horrible” (Minton & Carryer, 2005). They recalled undergoing procedures and treatment against their will, and some recalled fighting the nurses who performed them (Alexandersen et al., 2019; Ballard et al., 2006; Minton & Carryer, 2005). Moreover, a few patients recalled feeling scared when unfamiliar nurses carried out procedures on them (Löf et al., 2006).

### 3.4 Memories of being vulnerable and close to death

Patients described memories of being confronted with their vulnerability and dependency, giving them a sense of losing control over their situation. Patients remembered not being able to move, feeling thirsty and feeling dismayed by significant muscle loss. They recalled discomfort and shame related to the fear of not controlling the bowel function and being dependent on others to accommodate their needs (Alexandersen et al., 2019; Meriläinen et al., 2013; Page et al., 2019). Others remembered it as difficult to be awake during mechanical ventilation as it increased their awareness of the severity of their illness (Laerkner et al., 2017). “I don't quite understand why I had to be awake while I was so sick. I just don't. They said it would be better this way, but I found it hard. When my brain was so affected … I couldn't stand it. I felt like a fish out of water” (Laerkner et al., 2017).

Patients described feeling vulnerable when the medications made them feel hazy and the surroundings blurred. Furthermore, some recalled how they could not keep track of events and how they felt out of control when they were unable to retain information (Ballard et al., 2006). A few remembered how they felt trapped and trying to break free from the lines and equipment in an attempt to regain control (Löf et al., 2006; Minton & Carryer, 2005; Tembo et al., 2012).

The patients' difficulty communicating caused them to be robbed of their independence. They remembered feeling distressed and very frustrated because of the endotracheal tube hindering them in vocalizing their needs (Magarey & McCutcheon, 2005; Olsen et al., 2017; Tembo et al., 2012). In addition, some had problems understanding the nurses. Patients described distress in regard to the ever‐changing staff and several speaking foreign languages, thus putting additional pressure on communication (Laerkner et al., 2017; Löf et al., 2006; Magarey & McCutcheon, 2005; Olsen et al., 2017). These challenges made patients feel discouraged. The great strain the deficit in communication represented was made clearer when patients described memories of relief when regaining the ability to express themselves; “The use of a one‐way valve was an enormous relief and was described as ‘winning the Lottery.’ Being able to communicate provided a measure of control in an uncontrollable world” (Olsen et al., 2017). Patients recalled how calm and relieved they felt when nurses understood their gestures and could read their lips (Löf et al., 2006).

Patients remembered how they felt that death was imminent, causing distress and anxiety (Löf et al., 2008; Meriläinen et al., 2013; Tembo et al., 2012). They recounted episodes of horrifying and intense pain, causing great fear (Löf et al., 2006; Magarey & McCutcheon, 2005). The remembered pain was described by some as very distressing (Adamson et al., 2004; Alexandersen et al., 2019; Meriläinen et al., 2013) and all‐consuming: “They asked me if I had any pain and where the pain was… I could not say; the pain was everywhere” (Meriläinen et al., 2013). The sense that their life was in danger became manifest, especially in respect to ventilator treatment, and patients recalled panic and fear when they felt like they were not getting sufficient air supply (Karlsson & Forsberg, 2008; Löf et al., 2008; Storli et al., 2008; Tembo et al., 2012). These memories were strong and upsetting; “… I can remember feeling that I wasn't getting enough breath. I thought I was going to suffocate. My fighting spirit kicked in. I tried to pull it out [tube] … You get a feeling of how you might simply die!” (Tembo et al., 2012). The patients' confrontation with their own mortality was also outlined in how they felt exhausted from the treatment, wanting to give up (Löf et al., 2008; Magarey & McCutcheon, 2005). Despite a high symptom burden and severe medical condition, some patients had no recollections of thinking about death or questioning their survival. They remembered fighting for their recovery (Alexandersen et al., 2019).

## 4 DISCUSSION

This qualitative SR aimed to identify and synthesize the evidence regarding adult patients' memories from their stay in the ICU. Essentially, the majority of patients' memories were related to negative experiences and feelings from their stay in the ICU, and a range of memories confronted patients with life and death. In contrast, we found some positive memories regarding safety, secure and good care provided by nurses, who also were competent and collaborative.

The patients ‘confusion and disorientation of time and place, in addition to being aware of their inability to comprehend what was going on around them, seemed to evoke feelings of fear and reinforce the feelings of helplessness. These findings are in line with a study discovering that patients recalled the chaos and disorientation as the biggest threat in the ICU (Maddox et al., 2001). Therefore, patients could have a great need for knowing what was happening to them, and what was real versus unreal (Hupcey, 2000).

Our findings provide deeper insight into the contents of delusional memories generating intensity and level of fear as well as realistic and threatening nightmares. In addition, we found that most patients recalled that delusions evoked strong emotions, which seems to be consistent with what Storli et al. (2008) contended, the emotional intensity of the delusion is decisive for how firmly the delusion is rooted in the memory. Furthermore, several of the recalled delusions reflected a blurred border between the real and unreal. A review found that experiencing unpleasant events could be misinterpreted by the patients and easily change into delusions and nightmares (Kiekkas et al. 2010). This was displayed in our findings showing that nurses’ care and procedures could manifest as threatening nightmares, such as patients being convinced that they were being held captive or that the nurses were trying to kill them. Patients delusional memories from the ICU were associated with the development of diverse aspects of psychological distress, including feelings of anxiety and depression, problems sleeping and PTSD (Kiekkas et al., 2010). However, Aitken et al. (2016) found that it did not matter whether a memory was of a real event or a delusion; the negative outcomes were linked to the fact that the memory was distressing. Jones et al. (2001) strengthened this theory by linking PTSD to the number of adverse memories recalled from the ICU, not necessarily limited to the distressing delusional memories. Nevertheless, delusions and scary recollections seem to cause increased distress post‐ICU‐discharge. Our findings outline the strong presence of these potentially harmful memories in former ICU patients.

Our findings suggest that patients recalled both positive and negative emotions related to the care provided in the ICU, which reflect the complicated relationship between nurses and patients. Delmar (2012) claims that all people are fundamentally dependent on one another. Because we bestow our reality on each other, we surrender ourselves to a dependent relationship when interacting. By influencing each other's frame of mind, our actions and exuded emotions can either expand or constrict the other person's room of action. A person's opportunity to self‐express depends on how one is considered by others. According to Delmar (2012), the relationship between a nurse and a patient is not fully voluntary and reciprocal, making it an asymmetrical professional relationship based on dependency and power.

Some patients' room of action seemed to be provided by ICU nurses. How patients relied on nurses to initiate communication when they were intubated, and how patients recalled nurses talking over their heads, made them feel ignored and that the nurses did not have time for them. Nevertheless, ICU nurses may consider the patient and the surrounding equipment as a whole, which may lead to objectifying the patient (Almerud et al., 2007). Consequently, some of the recalled experiences of powerlessness we described in our findings may be due to nurses being ignorant or unaware of their power over the patient. Nurses need to expose the unconscious and invisible actions of power to prevent the constriction of patients (Delmar, 2012). Some of the nurses who attempted to help patients seemed to have had their actions interpreted by patients as an exercise of power. Patients recalled several of the procedures performed by nurses as threatening and harmful, episodes of oppression and actions being forced on them. Lykkegaard and Delmar (2013) question whether the context of life and death in the ICU camouflages the nurses' power actions by making them easily overlooked because of the fact that they do save lives. Our results increase the awareness of how great the impact an unbalanced power relationship can be on patients' sense of being in control.

Our findings show how nurses can contribute to patients' needed security as well as helplessness and fear, depending on how they distribute their power in care. Patients can be dependent and still be in control as long as their needs are met (Boggatz et al., 2007). Furthermore, our data suggest that some patients remembered empowering experiences when needing help. They recalled feeling great relief when their needs were met and did not seem to feel powerless. Our findings are substantiated by the study of Croxall et al. (2014) who emphasized how an effective communication between the caregiver (nurse), and the ICU‐patient was paramount in promoting long‐term psychological well‐being. In addition, the patients' memories of nurses providing safety and comfort care illustrate a well‐balanced power relationship where trust is established and is empowering to the patients. ICU patients may have an overwhelming need to feel safe (Hupcey, 2000). In our SR, some patients recalled nurses making them feel safe in the ICU environment, and their presence was recalled by several as calming and relaxing. These memories suggest that nurses can provide support and enhance patients' sense of independence during their stay in the ICU, regardless of the patients' level of dependency. The significance of a trusting relationship with the nurses is to ICU patients by its great impact on the patients' experiences in the ICU. ICU nurses have an important role in providing patients with safety by correctly handling their superiority in the unbalanced power relationship.

Our findings suggest that patients seemed to have strong memories of the sensation of death being imminent in the ICU as an existential threat continuously surrounding them. This was remembered as specific events such as experiencing not getting a sufficient air supply or witnessing other patients being critically ill, as well as an overall realization of the severity of their critical illness. These memories highlighted their experience of being vulnerable in the ICU, which seemed to make their proximity to death more tangible. This may be related to patients' high degree of physical and emotional dependency in the ICU (McKinley et al., 2002). Patients' memories of losing control and becoming increasingly vulnerable were particularly evident in memories of their restricted ability to communicate caused by the endotracheal tube. They also struggled to deal with the realization of being helpless and the fear of never regaining their independence. The negative emotions derived from being dependent and helpless in the ICU may be connected to the great value independence has to people in Western culture suggesting that when we lose our independence, our entire existence is at stake (Lykkegaard & Delmar, 2013). It is worth noting that patients who “nearly died” in the ICU raised questions about their entire existence after surviving (Parker, 1999).

### 4.1 Strengths and limitations of the review

A strength of this SR was that systematic searches were conducted in four databases, in addition to manual searches, and that study selection, quality appraisal and data extraction were performed by pairs of authors independently. The first author works as an ICU nurse, two other authors have worked as ICU nurses, while the last author has no such experience. The first author's pre‐understanding was discussed throughout the process and its influence with the last author considered, in an effort to set aside bias and make it possible to work past the pre‐understanding and discover new knowledge. The analysis was an interactive process where the first author analysed the data, while the last author asked critical questions to facilitate competing interpretations. The process of categorizing and the abstraction and interpretation into themes were discussed among all the authors, and revisions were made.

By limiting the systematic search to publications written in English or Scandinavian languages, studies that could help refine the knowledge of patients' memories from the ICU may have been excluded. The search terms may have limited the results and thereby limited the explored memories. Due to these choices, we may have introduced selection bias. As this is a review of qualitative evidence and the reviewers were not primary investigators of the included studies, there might be nuances and subtleties that were not picked up that could have enriched and deepened the findings. Nevertheless, the findings from our SR align with and reinforce the findings of the papers included.

Another limitation is related to the quality of the included studies; several studies did not state the researcher's context. Considering the validity of their results is difficult when the researcher's inevitable influence was not described. This implies that the findings presented in this review should be interpreted with some caution and might have limited transferability.

## 5 CONCLUSION

The unbalanced power relationship between nurses and the patients has potential to cause considerable distress for ICU patients. Our findings highlight patients' dependency on nurses in the ICU and a high level of vulnerability. The majority of memories brought forth feelings of fear, whether of loss of control, the imminence of death or powerlessness. Memories of confusion and surreal delusions were prominent. The complicated relationship between care giver and care receiver, and its many facets are made prominent in our study. The importance of a reassuring relationship between nurse and patient was highlighted by the recollection of contrasting emotions of dependency and fear in relation to the provided care.

All the studies were from high‐income countries, and although patient memories might be similar in other settings, future research is needed in other socioeconomic settings to explore the findings and to broaden our understanding.

## CONFLICT OF INTEREST

We have no conflict of interest.

## AUTHOR CONTRIBUTIONS

CCMM, MTS, MHL and SAS: Substantial contributions to conception and design, or acquisition of data, or analysis and interpretation of data, drafting the manuscript or revising it critically for important intellectual content, final approval of the version to be published. Each author should have participated sufficiently in the work to take public responsibility for appropriate portions of the content, agreed to be accountable for all aspects of the work in ensuring that questions related to the accuracy or integrity of any part of the work are appropriately investigated and resolved.

## ETHICAL APPROVAL

Ethical approval was not required since this study is a systematic review.

## Data Availability

Data availability is not relevant, since all data are available in original articles.
